# NSAIDs: Old Drugs Reveal New Anticancer Targets

**DOI:** 10.3390/ph3051652

**Published:** 2010-05-25

**Authors:** Gary A. Piazza, Adam B. Keeton, Heather N. Tinsley, Jason D. Whitt, Bernard D. Gary, Bini Mathew, Raj Singh, William E. Grizzle, Robert C. Reynolds

**Affiliations:** 1 Southern Research Institute, 2000 9^th^ Avenue South, Birmingham AL, 35205, USA; 2 The University of Alabama at Birmingham, 703 19^th^ Street South, Birmingham AL, 35294, USA; 3 Vivo Biosciences Inc., 1601 12^th^ Avenue South, Birmingham AL, 35205, USA

**Keywords:** NSAIDs, sulindac, cancer, colon, chemoprevention

## Abstract

There is compelling evidence that nonsteroidal anti-inflammatory drugs (NSAIDs) and cyclooxygenase-2 selective inhibitors have antineoplastic activity, but toxicity from cyclooxygenase (COX) inhibition and the suppression of physiologically important prostaglandins limits their use for cancer chemoprevention. Previous studies as reviewed here suggest that the mechanism for their anticancer properties does not require COX inhibition, but instead involves an off-target effect. In support of this possibility, recent molecular modeling studies have shown that the NSAID sulindac can be chemically modified to selectively design out its COX-1 and COX-2 inhibitory activity. Unexpectedly, certain derivatives that were synthesized based on *in silico* modeling displayed increased potency to inhibit tumor cell growth. Other experiments have shown that sulindac can inhibit phosphodiesterase to increase intracellular cyclic GMP levels and that this activity is closely associated with its ability to selectively induce apoptosis of tumor cells. Together, these studies suggest that COX-independent mechanisms can be targeted to develop safer and more efficacious drugs for cancer chemoprevention.

## 1. Introduction

Epidemiological, clinical, and preclinical studies provide compelling evidence that NSAIDs, including COX-2 selective inhibitors, have antineoplastic properties. Population-based studies have shown that chronic use of NSAIDs is associated with reduced incidence of colorectal cancer by as much as 30-50% [1,2]. Moreover, clinical studies have shown that certain NSAIDs can reduce precancerous adenomas in patients with familial or sporadic adenomatous polyposis. For example, the non-selective COX inhibitor sulindac and the COX-2 selective inhibitor celecoxib have been shown to prevent the formation and cause regression of adenomas in patients with familial polyposis [3,4,5,6,7,8]. Unfortunately, COX-1 and/or COX-2 inhibition and depletion of physiologically important prostaglandins is associated with gastrointestinal, renal and cardiovascular toxicities that limit the use of NSAIDs and COX-2 inhibitors for cancer chemoprevention [9,10,11]. Aside from toxicity, another major limitation is that currently available anti-inflammatory drugs do not completely protect against disease progression. For example, case reports have described individuals with adenomas who developed colorectal cancer despite long term treatment with sulindac [3,12]. 

Numerous studies have shown that cyclooxygenase enzymes are overexpressed and prostaglandin levels are increased in various tumor types. These observations support the commonly held view that inflammation plays an important role in tumorigenesis and that suppression of prostaglandin synthesis is responsible for the chemopreventive activity of NSAIDs [13,14,15,16]. However, there is opposing evidence to suggests that a COX-independent mechanism is either responsible for or contributes to the antineoplastic properties of NSAIDs [17,18,19,20]. Given the toxicity limitations of currently available NSAIDs, the elucidation of the biochemical and cellular pathways that are responsible for their antineoplastic activity could provide insight to alternative pathways that could be targeted to discover and develop potentially safer and more efficacious drugs for cancer chemoprevention. Here we review evidence that the cancer chemopreventive properties of NSAIDs are COX-independent, including recent studies that have shown that the NSAID, sulindac can be chemically modified to reduce COX binding, while enhancing tumor cell growth inhibitory activity. Others studies have suggested a mechanism involving cyclic guanosine monophosphate phosphodiesterase inhibition, which is also reviewed.

## 2. Colorectal Cancer

Colorectal cancer is the 3^rd^ most common malignant disease in Western countries [21]. In the United States, colorectal cancer is a major public health problem that accounts for 12% of all newly diagnosed cancers [22]. Approximately 6% of cancer deaths each year are from colorectal cancer [23]. Those individuals who have a genetic predisposition are at higher risk for developing colorectal cancer and will develop precancerous lesions at an earlier age compared with the general population. For example, patients with familial adenomatous polyposis (FAP), a rare autosomal dominant disease, can develop hundreds to thousands of precancerous adenomas by adolescence and will inevitably develop colon cancer unless the colon is surgically removed [24]. A larger percentage of the population develops sporadic adenomas, which involves the same genetic defect as in FAP patients. Despite a significant reduction of colorectal cancer incidence by endoscopic surveillance, colonoscopy is an invasive procedure that is associated with significant morbidity. In addition, the colon mucosa contains precancerous cells that reside in microscopic lesions referred to as aberrant crypt foci, which are found in the uninvolved colon epithelium in patients and cannot be detected by conventional colonoscopy. As such, there is a clear unmet medical need to develop new drugs that could be used in conjunction with colonoscopy to reduce colon cancer progression in moderate to high risk individuals. 

The majority of colorectal cancers are caused by mutations in the *APC* (adenomatous polyposis coli) gene, which is considered to be the earliest genetic defect in the aberrant crypt foci-adenoma-carcinoma sequence [25]. Germ-line mutations in a single allele of the *APC* gene are sufficient to cause the formation of adenomas that progress to adenocarcinomas as colonocytes develop a second mutation in the wild-type *APC* allele as well as in other oncogenes and tumor suppressor genes involved in colorectal carcinogenesis [26]. The wild-type APC protein is expressed in non-proliferating epithelial cells of colonic crypts where it functions as a member of a supra-molecular complex with axin and glycogen synthetase kinase 3β to maintain low levels of β-catenin by regulating ubiquitination and proteosomal degradation [27]. The loss of APC protein by mutations provides a growth advantage to colonocytes to stabilize β-catenin, which is an important transcription factor with oncogenic properties. In neoplastic cells of the colon, β-catenin levels are high in the nucleus where it plays an essential role in the synthesis of tumor cell growth promoting and survival proteins such as cyclin D and survivin [28]. 

## 3. Cancer Chemopreventive Activity of NSAIDs

Numerous population-based studies that have surveyed individuals who regularly take NSAIDs provide strong evidence that long-term use of this widely prescribed class of drugs can significantly reduce the risk of death from colorectal cancer by as much as 50% [29]. Additionally, treatment with certain high potency NSAIDs such as sulindac can reduce the number and size of adenomas in patients with familial or sporadic polyposis [6]. Experimental studies support these observations by showing the capacity of various NSAIDs to inhibit colon carcinogenesis in rodent models with *APC* mutations or involving chemically-induced colon tumorigenesis [30,31], including models of aberrant crypt foci formation. Recent studies have shown that certain NSAIDs can suppress β-catenin levels in the nucleus to inhibit transcriptional activity [32]. Although the underlying molecular target(s) responsible for the antineoplastic activity of NSAIDs has been elusive as discussed below, the suppression of β-catenin mediated transcription by NSAIDs may provide a highly specific mechanism to compensate for the genetic aberrations that are associated with *APC* mutations, which are ultimately responsible for the initiation of colorectal cancer.

## 4. Classification of NSAIDs

**Figure 1:**
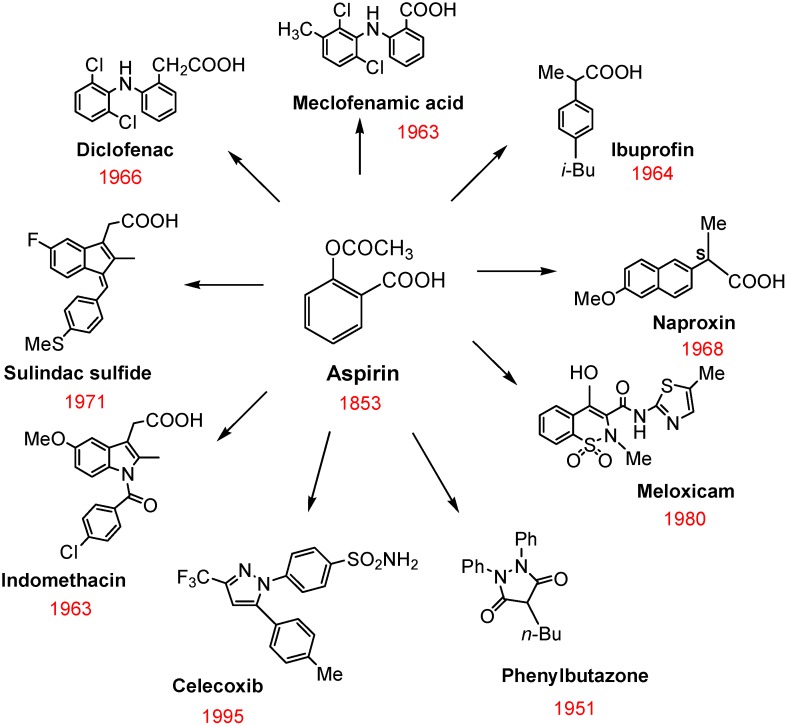
Chemical structures of commonly used NSAIDs.

NSAIDs are a chemically diverse family of drugs that are available over-the-counter or by prescription and are commonly used in acute situations to treat a variety of inflammatory conditions or chronically to manage pain associated with arthritis. Figure 1 shows the chemical structures of the most widely used NSAIDs, which remarkably still includes aspirin that was discovered over 150 years ago. The pharmacological basis for the anti-inflammatory activity of NSAIDs involves the inhibition of COX isozymes and blockage of the conversion of arachidonic acid to prostaglandin H_2_, a precursor for the synthesis of prostaglandins, prostacyclins, and thromboxanes [9]. Two distinct COX isozymes have been characterized that share similar catalytic activity, but have different modes of expression and sensitivity to inhibitors [10]. COX-1 is a constitutive enzyme responsible for the regulation of prostaglandin biosynthesis in normal tissues and serves an important role in maintaining blood flow necessary for gastric cytoprotection and renal homeostasis. As such, the chronic administration of non-selective COX inhibitors is associated with risk of gastrointestinal and renal toxicity. 

The COX-2 isozyme is inducible and expressed in inflammatory cells and cancer cells and was considered to be an ideal drug target for inhibiting inflammation and tumorigenesis. However, recent clinical studies have shown an association between the use of COX-2 selective inhibitors such as rofecoxib (Vioxx®) and celecoxib (Celebrex®) and increased incidence of stroke and myocardial infarction. These toxicities are believed to be attributed to COX-2 inhibition and the suppression of endothelial-derived prostacyclin levels that function as a vasodilator and inhibitor of platelet aggregation [33]. Vioxx was withdrawn from the market, while Celebrex remains available, despite its associated risk of cardiovascular toxicity. 

## 5. Cyclooxygenase Independent Anticancer Activity of NSAIDs

Numerous investigators have suggested that a COX-independent effect may either contribute to or be fully responsible for the chemopreventive properties of NSAIDs, as well as COX-2 selective inhibitors [17,18,19,20,34,35,36]. For example, NSAIDs and COX-2 selective inhibitors can suppress the growth of tumor cells that do not express COX-2 [20], while supplementation with exogenous prostaglandins does not reverse the growth inhibitory activity of NSAIDs [37]. Additionally, the rank order potency among NSAIDs to inhibit prostaglandin synthesis does not match the potency to inhibit tumor cell growth [38]. In general, appreciably higher dosages of NSAIDs are required to inhibit tumor cell growth compared to anti-inflammatory dosages [39]. For example, a series of chemically diverse NSAIDs as listed in Table 1 are ranked based on their potency to inhibit colon tumor cell growth *in vitro* and their potency to inhibit either COX-1 or COX-2. In addition to the lack of correlation between these two activities, the concentration range required to inhibit tumor cell growth significantly exceeds the concentrations required to inhibit COX-1 and COX-2. Moreover, the concentration range required to inhibit tumor cell growth exceeds the concentration in blood that can be achieved in humans with standard dosages. Still, NSAIDs show evidence of chemopreventive efficacy in long term studies, which may be the consequence of chronic administration. As highlighted, aspirin may be unique among other NSAIDs given its ability to irreversibly bind COX, which may provide a sustained anti-inflammatory benefit. 

**Table 1:** Tumor cell growth and COX inhibitory activity of a panel of NSAIDs.

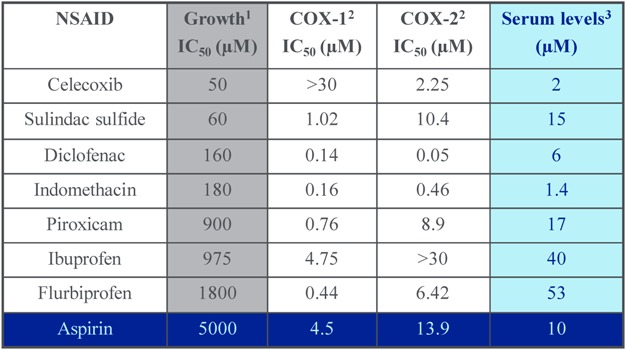

^1^ Human HT29 colon cells, 72 hrs, MTS assay [unpublished data]; ^2^ Whole blood COX assays [40]; ^3^ Therapeutic dosages [41].

Perhaps the most compelling evidence for a COX-independent mechanism comes from studies showing that certain chemically related derivatives, which include metabolites (e.g. sulindac sulfone or exisulind), *R*-enantiomers (e.g. *R*-flurbiprofen, *R*-etodolac), and synthetic derivatives (e.g. OSU-03012, a celecoxib derivative; SRI 21009, a sulindac derivative) retain anticancer activity, despite lacking COX inhibitory activity [42,43,44]. 

## 6. Cancer Chemopreventive Activity of Sulindac and Its Non-COX Inhibitory Sulfone Metabolite

**Figure 2:**
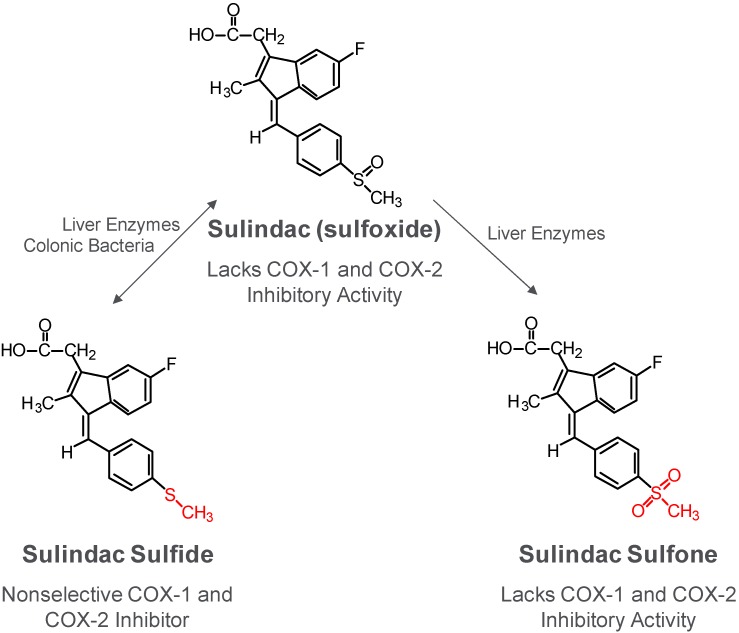
Metabolism of sulindac.

Sulindac is considered to be the most effective among the NSAIDs and COX-2 inhibitors with regard to cancer chemoprevention. For example, clinical trials involving patients with familial adenomatous polyposis showed that six months of treatment with celecoxib at a dose of 800 mg per day caused a 28% mean reduction in polyp number [8]. By comparison, sulindac reduced polyp number and size by approximately 60-70% at a daily dose of 300 mg [3,6,7]. In addition, there is an abundance of evidence showing strong cancer chemopreventive efficacy of sulindac in a variety of animal models at dosages significantly less than those required for celecoxib [45,46]. As shown in Figure 2 and reviewed previously [47], sulindac is a prodrug that requires reductive metabolism of the sulfoxide by colonic bacteria to a sulfide, which is a non-selective COX inhibitor that is responsible for the anti-inflammatory activity of sulindac. A sulfone metabolite is also generated by oxidation of the sulfoxide in the liver, but does not have anti-inflammatory activity. The reduction of the sulfoxide to the sulfide is reversible, while the oxidation of the sulfide or sulfoxide to the sulfone is irreversible.

Sulindac sulfone has been shown by several investigators to inhibit tumor cell growth and induce apoptosis *in vitro* in a number of tumor cell lines of diverse histological origin despite its lack of COX-1 and COX-2 inhibitory activity and can inhibit chemical-induced carcinogenesis of the colon, mammary glands, lung and bladder [18,30,34,48,49,50,51,52,53]. The inhibitory activity of sulindac sulfone on colon tumor formation induced in rats by the carcinogen, azoxymethane is summarized in Table 2 and has been previously published [30]. Although higher dosages were required, sulindac sulfone inhibited colon tumor formation to a level comparable to sulindac. Unlike sulindac, the antitumor effect of sulindac sulfone was not accompanied by a suppression of prostaglandin levels in the colon mucosa. The anticancer activity of sulindac sulfone in the azoxymethane-induced model of colon tumorigenesis was associated with plasma levels of the drug that exceeded concentrations required to inhibit tumor cell growth and induced apoptosis as observed *in vitro* using cultured colon tumor cell lines [18,34].

**Table 2:** Inhibition of colon tumorigenesis in rats by sulindac sulfone and sulindac.

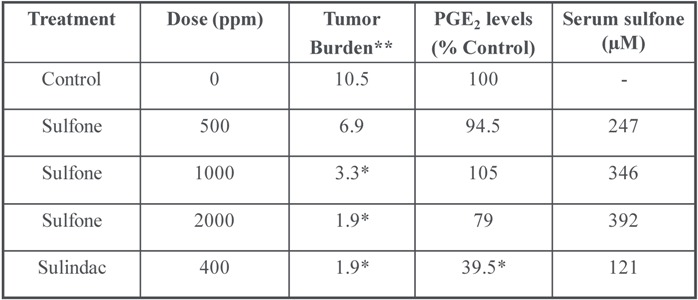

* *P* < 0.05; ** Sum of sizes (cm) of adenomas and adenocarcinomas.

In clinical trials, sulindac sulfone was shown to cause significant adenoma regression in patients with familial [54] or sporadic [55] adenomatous polyposis but did not receive FDA approval due to hepatotoxicity. Nonetheless, an interesting observation from the early clinical studies of sulindac sulfone in familial adenomatous polyposis patients is that treatment was associated with an increase the percentage of apoptotic cells in adenomas, but not in the normal colonic mucosa. A summary of these results are shown in Figure 4 and have been previously published [54]. These observations suggest that the underlying biochemical mechanism responsible for the anticancer properties of sulindac sulfone involve a selective effect on neoplastic cells that is COX-independent. Despite its shortcomings in potency and toxicity, sulindac sulfone provided important proof-of-concept evidence to support further research on the underlying mechanism of action.

**Figure 3:**
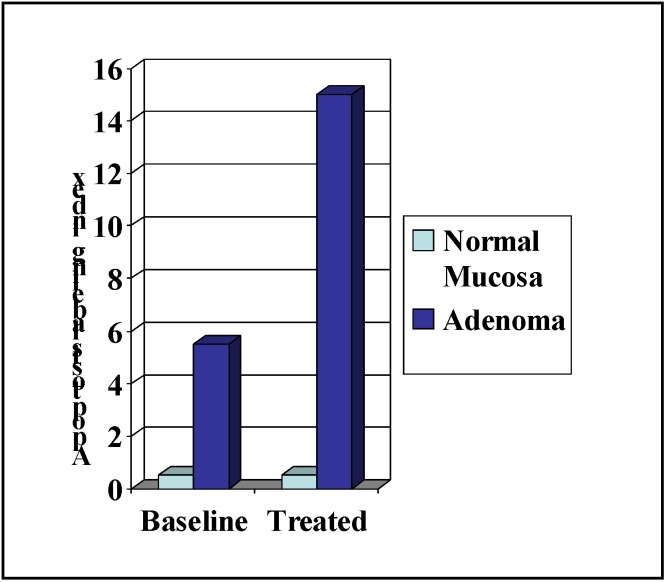
Apoptosis induction by sulindac sulfone in FAP patients.

## 7. Design of a Novel Non-COX Inhibitory Sulindac Derivative with Potent Tumor Cell Growth Inhibitory Activity

**Figure 4:**
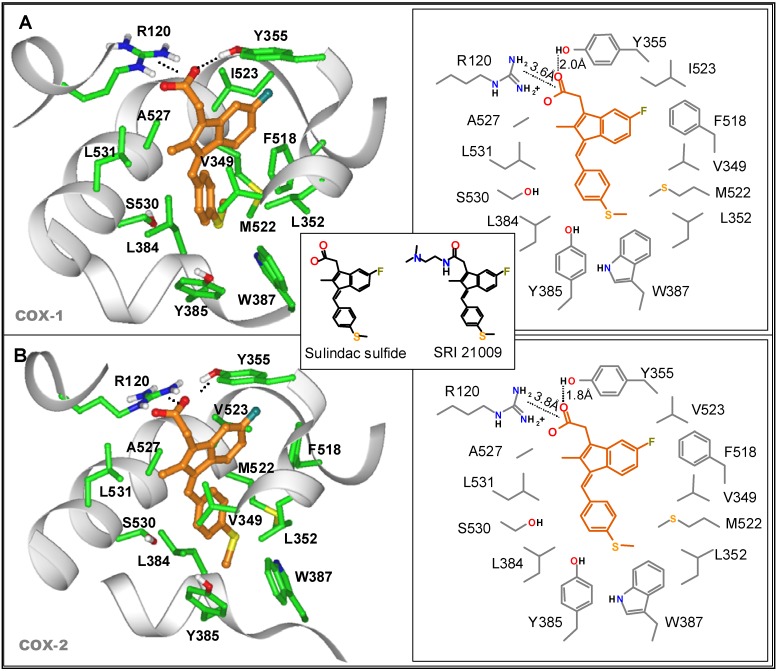
Molecular modeling of SS binding to COX-1 (A) or COX-2 (B). Chemical structures of SS and SRI 21009 (SSA) are shown in the insert.

To develop new drug candidates with improved potency, more recent studies have focused on strategies to design out the COX inhibitory activity of the more potent sulfide metabolite of sulindac. As recently reported [56], *in silico* modeling studies that docked sulindac sulfide (SS) into its binding domain on COX isozymes were conducted using the published structures of COX-1 and COX-2 (PDB entries 2OYE and 6COX, respectively) obtained from X-ray diffraction studies. The orientation of SS bound to COX-1 and COX-2 was found to be very similar as shown in Figure 4. It was also noted that the carboxylic acid moiety on SS formed a salt bridge with the positively charged guanidinium moiety of arginine 120 (R120) that was common to both COX-1 and COX-2, which constrains the rest of the molecule in a region that is rich in non-polar amino acids.

The observations suggested the synthesis of a series of SS derivatives with amide substitutions in place of the carboxylic acid. Following screening for tumor cell growth and COX inhibitory activity, a subset of analogs containing a basic amino group was unexpectedly found to inhibit colon tumor cell growth with high potency. A derivative with a *N,N*-dimethylethylamine group as shown in Figure 4 (insert) was among the most potent inhibitors identified and was studied in more detail. This derivative was designated as SRI 21009 and is referred to here as sulindac sulfide amide (SSA) for simplicity. Using purified COX-1 and COX-2 isozymes, SS was found to inhibit COX-1 and COX-2 with IC_50_ values of 1.8 and 6.3 μM, respectively. By comparison, SSA did not inhibit COX-1 at concentrations as high as 300 μM and only weakly inhibited COX-2 with an IC_50_ value of 164.5 μM; a level unlikely to be pharmacologically relevant. 

**Figure 5:**
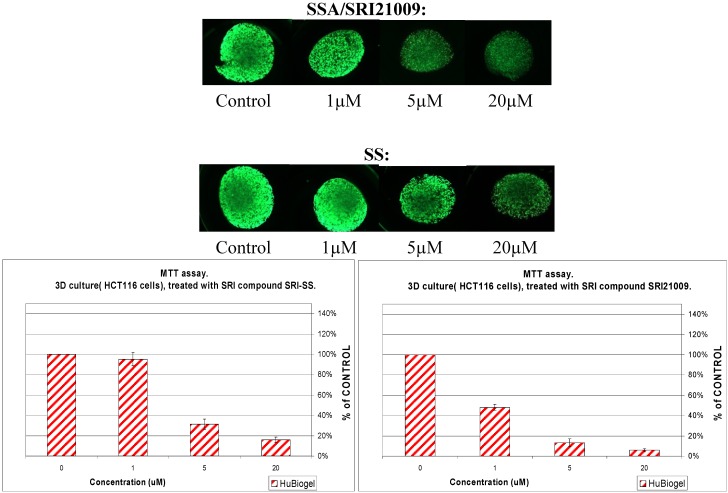
Inhibition of human HCT116 colon tumor cell growth in a 3-dimensional spheroid model by sulindac sulfide and sulindac sulfide amide (SRI 21009).

In addition to *in vitro* tumor cell growth inhibitory activity using colon tumor cell lines with IC_50_ values of approximately 2 µM as previously published [56], SSA was also evaluated for anti-tumor efficacy using a three dimensional mini-tumor assay model that emulates *in vivo* like multi-cellular tumor growth and biology as described previously [57]. Viable mini-tumors (1 mm size) of HCT-116 colon cancer cells were generated using a 3D human Biogel® culture system (HuBiogel, Vivo Biosciences Inc.). As shown in Figure 5, SSA treatment significantly inhibited colony formation in a dose-dependent manner with ~80% inhibition at 5 µM. By comparison, sulindac sulfide was appreciably less effective, causing no inhibition at 1 µM and ~40% inhibition at 5 µM which was equivalent to 1 µM SSA. 

The apoptosis inducing properties of SSA and *in vivo* anticancer activity in the HT-29 colon tumor xenograft model has been previously reported as well as studies showing combination benefits with conventional chemotherapeutic drugs, such as irinotecan [56]. Together, these studies suggest that SSA or analogs with improved selectivity, potency, and bioavailability have the potential to be more efficacious and less toxic than sulindac for cancer chemoprevention, which merit further preclinical development.

## 8. COX-Independent Targets of NSAIDs

**Figure 6:**
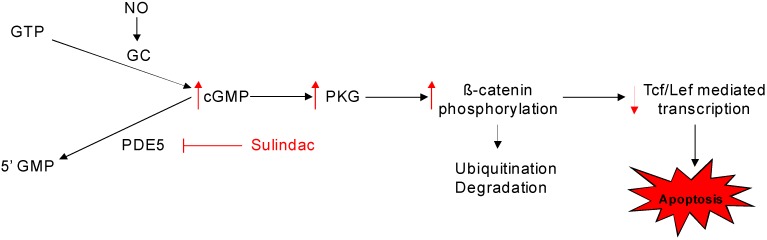
Mechanistic model for the apoptosis inducing properties of sulindac.

A number of COX-independent targets have been proposed to mediate the cancer chemopreventive properties of NSAIDs, including 15-lipoxygenase [58], Ras [59], PPAR [60], NF-κB [61], PDK-1/Akt [62], phosphodiesterase [36,63] as well as others [64,65]. A comprehensive review of the potential non-COX targets of NSAIDs has been previously published [66]. As reported previously sulindac sulfone can inhibit cyclic guanosine monophosphate phosphodiesterase (cGMP PDE) and that this activity correlated with potency to inhibit colon tumor cell growth among a series of sulindac analogs [63]. These studies provided initial evidence that cGMP PDE may represent an important non-COX target that is responsible for the tumor cell growth inhibitory and apoptosis inducing activities of sulindac. In support of this possibility, recent studies have shown that SS can selectively inhibit the cGMP specific PDE5 isozyme and that cGMP elevation is closely associated with its tumor cell growth inhibitory and apoptosis inducing activity [67]. In addition, PDE5 has been found to be overexpressed in various carcinomas and appears to be the predominant cGMP degrading isozyme in numerous tumor cell types. Based on these and other studies, a mechanistic model for the apoptosis inducing properties of sulindac as shown in Figure 6 can be envisioned, which involves PDE5 inhibition, cGMP elevation, and protein kinase G activation. As previously reported, β-catenin is a potentially important substrate of protein kinase G to induce degradation, thereby suppressing the transcription of important growth regulatory genes such as cyclin D and survivin that are necessary for tumor cell proliferation and survival [63,68,69,70]. Conventional PDE5 inhibitors such as sildenafil that are used for treating erectile dysfunction, however, do not inhibit tumor cell growth as does SS. While such drugs are capable of inhibiting PDE5 activity in lysates from tumor cells, they are unable to induce cGMP levels or activate cGMP signaling in cancer cells. The lack of anticancer activity of sildenafil and other conventional PDE5 inhibitors may be attributed to efflux mechanisms involving membrane transport proteins, although additional studies are required to investigate this and other possibilities. 

## 9. Conclusions

It has been estimated that 28 million people in the US develop sporadic colonic adenomas [71]. Approximately 10% form lesions at a high rate and could benefit from a safe and efficacious chemopreventive drug that would serve as an adjunct with colonoscopy to prevent new lesions from forming and reducing the overall risk of disease progression. NSAIDs have shown promising activity in experimental, clinical and epidemiological studies, although gastrointestinal, renal, and cardiovascular toxicity resulting from the suppression of cyclooxygenase-1 and/or -2 and the depletion of physiologically important prostaglandins limits their use for chemoprevention. Mechanistic studies suggest that a COX-independent or off-target effect, possibly involving PDE5 inhibition and cGMP elevation to induce apoptosis may contribute to their antineoplastic properties. Therefore, it may feasible to develop safer and more efficacious drugs for chemoprevention by targeting PDE5, although further studies are necessary to determine the exact role of this isozyme and the cGMP pathway in tumorigenesis. 
